# Multiple drug resistance due to resistance to stem cells and stem cell treatment progress in cancer (Review)

**DOI:** 10.3892/etm.2014.2141

**Published:** 2014-12-16

**Authors:** CHONG DI, YAODONG ZHAO

**Affiliations:** Department of Neurosurgery, Shanghai 10th People’s Hospital, Tongji University School of Medicine, Shanghai 200072, P.R. China

**Keywords:** cancer stem cell, multiple drug resistance, adenosine triphosphate-binding cassette transporter, aldehydes dehydrogenase, microenvironment, epithelial to mesenchymal transition

## Abstract

In recent years, the cancer stem cell (CSC) theory has provided a new angle in the research of cancer, and has gradually gained significance. According to this theory, the multiple drug resistance (MDR) of cancer is most likely due to the resistance of CSCs, and a significant quantity of research has been carried out into the MDR mechanisms of CSC. Over time, some of these mechanisms have been gradually accepted, including ATP-binding cassette transporters, aldehyde dehydrogenase, the CSC microenvironment and epithelial to mesenchymal transition. In the present review, we summarize these mechanisms in detail and review possible appropriate therapy plans against CSCs based on CSC theory.

## 1. Introduction

Cancer is a notable threat to human health with a rising incidence rate; however, there is still no known cure. In recent years, the cancer stem cell (CSC) theory was proposed, which provided a new angle in the research of cancer, and gradually gained significance. According to this theory, the multiple drug resistance (MDR) of cancer is most likely due to the resistance of CSCs, which have received increasing attention from oncologists around the world (1). Therefore, we intend to review the common mechanisms of MDR in CSC, so as to discuss possible relevant therapeutic strategies against cancer.

## 2. Cancer stem cells

The concept of CSCs was first raised by Park *et al* (2) in 1971. The concept was that cancers are diseases driven by a subpopulation of self-renewing CSCs, which possess the ability to generate the diverse differentiated cell populations that comprise the cancer mass (3). Since then, CSCs have been identified and isolated from tumors of the hematopoietic system, breast, lung, prostate, colon, brain, head and neck, and pancreas.

Existing therapies have been developed largely against the bulk population of tumor cells to shrink tumors. However, most cancer cells only have limited proliferative potential; an ability to shrink a tumor mainly reflects an ability to kill these cells. Even therapies that cause complete regression of tumors might spare sufficient CSCs to allow regrowth of the tumors (4). Only therapies that are more specifically directed against CSCs might result in more durable responses and even a cure for metastatic tumors.

## 3. Mechanisms of CSC MDR

### ATP-binding cassette (ABC) transporters

The ABC transporter superfamily in humans includes at least 49 genes grouped into seven families (from A to G) with various functions, and at least 16 of these proteins are implicated in cancer drug resistance (5). They bind ATP and use the energy to drive the transport of various molecules across the plasma membrane; since they can eject anti-cancer drugs from cells, it may lead to the drug’s resistance (6). Among the ABC proteins, the most significant are glycoprotein P (P-gp), encoded by the ABCB1 [multidrug resistance protein 1 (MDR1)] gene, and breast cancer resistance protein (BCRP or ABCG2), which was cloned from a mitoxantrone-resistant subline of the breast cancer cell line MCF-7. BCRP confers resistance to a number of chemotherapeutics, including mitoxantrone. Other well-known proteins from the ABC family responsible for MDR are MDR-related protein (MRP) 1 (ABCC1) and MRP2 (ABCC2). ABC transporters are also expressed in normal stem cells to maintain a relatively stable intracellular environment and to keep them in a quiescent state. In addition, these transporters have certain other notable roles in normal physiology in the transport of drugs across the placenta and the intestine, and are essential components of the blood-brain and blood-testis barriers. By using the energy from ATP hydrolysis, these transporters actively efflux drugs from cells, serving to protect them from cytotoxic agents.

### Evidence for MDR of CSCs mediated by ABC transporters

Numerous studies have demonstrated the role of ABC transporters in CSC resistance. CD133-positive glioma stem cells exhibited a notable effect on tumor resistance to chemotherapy, which is possibly as a result of their high expression of ABCG2/BCRP1 (7). Similar results also were observed in the CSCs of lung cancer (8), osteoma sarcomatosum (9), ovarian cancer (10), prostatic carcinoma (11) and nasopharyngeal carcinoma (12). Furthermore, the high ABC transporter levels were associated with increased Akt signaling in drug-resistant CSCs, and the Akt signaling was able to alter the subcellular localization of BCRP transporters, thus determining drug efflux in CSCs (13). In the same study, Akt signal inhibition by P13K inhibitors not only suppressed cancer cell proliferation, but also increased the sensitivity of drug-resistant cells (13).

### Therapeutic measures targeting MDR mediated by ABC transporters

Theoretically, ABC transporter inhibitors could reverse the chemotherapy resistance of CSCs and eliminate tumors. Currently, its inhibitors have been developed to the third generation. First-generation compounds included drugs identified as ABCB1 inhibitors, including verapamil and cyclosporine, which were in clinical use to treat other diseases. These inhibitors were combined with a range of chemotherapy regimens for numerous types of cancer, but the results were not satisfactory. The clinical results for the second-generation inhibitors, including valspodar and biricodar, were also disappointing. Fumitremorgin C and its chemically synthesized derivatives including Ko143 have been developed; certain studies have demonstrated a positive effect *in vitro*, particularly for Ko143, which has high specificity and low toxicity (14). Studies with Ko143 have also revealed that inhibition of ABCG2 allows for a greater absorption of certain drugs across the intestine (14). In addition, the compound GF120918 is an ABCB1 inhibitor that has been demonstrated to inhibit ABCG2 *in vitro* as well as *in vivo* (15). Compared with those of previous generations, other inhibitors, including zosuquidar (LY335979) and tariquidar (XR9576), have higher inhibitory activity and selectivity, without affecting the metabolism of chemotherapeutic drugs themselves, and it is possible to overcome the resistance to CSCs. Moreover, they have already been approved for clinical studies. Certain drugs are in clinical use; for example, difluorinated curcumin enhances the sensitivity of CD44^+^CD166^+^ colon carcinoma stem cells to the combination of 5-fluorouracil and oxaliplatin by a mechanism that involves ABCG2 downregulation (15). The crude extract of traditional Chinese medicine *Schisandra* and its active ingredient deoxyschizandrin have the effect of reversing MDR (16), and enhance the curative effect of vincristine and adriamycin. *Schisandrae Lignans* inhibits the outflow of intracellular drugs (17,18). Chai *et al* (19) considered that schizandrin had strong potential in terms of reversing MDR, and that it could induce apoptosis and decrease the expression of P-gp and protein kinase C. Other medicines, including salinomycin (20,21), nigericin and abamectin have a similar effect. Nicardipine is also an inhibitor of ABCG2 (7).

### Aldehyde dehydrogenase (ALDH)

The ALDHs are a family of nicotinamide adenine dinucleotide phosphate [NAD (P)^+^]-dependent enzymes involved in detoxifying a wide variety of aldehydes to weak carboxylic acids, leading to increased protection of the cell against insult by environmental chemicals and drugs. ALDHs are also regarded as a type of biomarker for stem cells. They include the ALDH1 family (ALDH1A1, 1A2, 1A3, 1L1, 1L2), ALDH2 and ALDH3A1. In particular, ALDH1A1 and ALDH3A1 have been shown to play significant functional roles in stem cells (22), which was demonstrated in hematopoietic stem cells, neural stem cells and adipose tissue stem cells.

### Evidence for MDR of CSCs mediated by ALDHs

ALDH1 is a CSC marker and its presence is strongly correlated with tumor malignancy as well as self-renewal properties of stem cells in various tumors, including breast cancer, hepatoma, colon cancer and lung cancer (22). CSCs with a high expression of ALDH have the characteristics of being chemotherapy-resistant, which has been proven in breast cancer stem cells (23) and head/neck squamous cell carcinoma stem cells (24). The mechanism of ALDH expression of the gene may be associated with high Snail expression, since the knockdown of Snail expression significantly decreases the expression of ALDH1, inhibits cancer stem-like properties and blocks the tumorigenic abilities of CD44^+^CD24^–^ALDH1^+^ cells (24).

### Therapeutic measures targeting MDR mediated by ALDHs

A number of natural compounds are effective against CSCs and lead to decreasing numbers of ALDH-positive cells (25). For example, exposure of breast cancer cells to the dietary polyphenols, curcumin and piperine, severely reduces mammosphere formation (26), as do sulforaphane (27) and butein (28).

In addition, the role of chemical synthesis of the drug in inhibiting ALDH has also been studied. The specific ALDH inhibitor diethylaminobenzaldehyde may increase the sensitization of ALDH^bri^CD44^+^ cells to chemotherapy/ radiotherapy (23). Disulfiram is capable of inhibiting ALDH and enhancing the sensitization of cancer cells to chemotherapy (29). All-trans-retinoic acid treatment results in a 50–60% reduction in ALDH activity and proteins of both isozymes, ALDH1A1 and ALDH3A1, leading to the increased sensitization of ALDH1^+^ cancer cells to chemotherapy.

### Microenvironment

#### Mechanisms of the microenvironment influencing MDR of CSCs

Normal adult stem cells are regulated by molecular cues provided by neighboring connective tissue cells, mainly mesenchymal (fibroblast-like) cells and vascular cells; these stromal cells contribute to the stem cell niche. CSCs often appear to be present in and influenced by a similar microenvironment, which has been proven in brain cancer (30), squamous cell carcinoma (31), bladder cancer (32), leukemia (33,34) and colorectal cancer (35). The tumor microenvironment has been recognized as a major factor influencing the growth of cancer and impacting the outcome of therapy. Thus, niche cells have become an attractive target for chemotherapeutic agents (36). Targeted therapies through the production of secreted factors which might drive tumor growth and MDR may be available (37). Hypoxia has been considered as a major feature of the tumor microenvironment and a potential contributor to the MDR and enhanced tumorigenicity of CSCs (38). The acidic microenvironment around hypoxia cells is combined with the activation of a subset of proteases that contribute to metastasis (39). Due to aberrant angiogenesis and the inaccessible location, hypoxic cells are less likely to accumulate therapeutic concentrations of chemotherapeutics, which lead to MDR. Therefore, targeting the CSC niche in combination with chemotherapy may provide a promising strategy for eradicating CSCs.

#### Therapeutic measures targeting MDR mediated by the microenvironment

Treatment of animals with DC101, an antibody of the vascular endothelial growth factor (VEGF) receptor, leads to a transient increase in oxygenation and deeper penetration of molecules into experimental tumors. An increased response of tumors to combined treatment with chemotherapy and anti-angiogenic agents has been demonstrated (40). Bevacizumab, another type of antibody to VEGF receptor, was reported to have a similar effect (41). Agents that damage existing blood vessels in tumors may also influence the response to chemotherapy. Vascular disrupting agents (including tumor necrosis factor, flavone acetic acid and its derivatives, and tubulin-binding agents including combretastatin A-4 disodium phosphate) directly damage the established tumor endothelium and have been shown to increase vessel permeability and drug delivery (42). Certain other medicines, for example histamine, a selective endothelin receptor A antagonist and botulinum neurotoxin type A, increase tumor blood flow and have been shown to promote *in vivo* tumor perfusion and to delay tumor growth when combined with cyclophosphamide (10). However, there also is some dispute. Injecting vascular-disrupting agents prior to chemotherapy may be problematic since it could result in reduced blood flow and increased interstitial fluid pressure, which together could impair the delivery of drugs to tumors and lessen the curative effect (42).

#### Epithelial to mesenchymal transitions (EMT)

EMT is a process involving the dissolution of endothelial cell-cell junctions and loss of apico-basolateral polarity of endothelial cells, resulting in the formation of migratory mesenchymal cells with invasive properties. The loss of polarity and gain of motile characteristics of mesenchymal cells during embryonic development has prompted comparisons with metastatic cancer cells during malignant progression (43). Notably, these properties have also been ascribed to normal stem cells and CSCs. Cancer is a disease of abnormal wound healing and tissue repair, which is accompanied by pathophysiological EMT in adult tissues. Accordingly, EMT is able to trigger reversion to a CSC-like phenotype, providing an association between EMT, CSCs and MDR (44). In addition, a previous study has demonstrated that CSCs and EMT-type cells could play critical roles in drug resistance (45).

AMP-activated kinase (AMPK) inhibits EMT; however, the diabetes drug metformin may activate AMPK, which results in the elective killing of CSCs in combination with chemotherapy (46). However, it was unclear whether the positive results were a result of the combination with metformin. In addition, histone deacetylase (HDAC) is another significant molecule in EMT, and HDAC promotes EMT by inhibiting E-cadherin (47). Certain HDAC inhibitors, including trichostatin A and vorinostat, induce the differentiation of mesenchymal-like cancer cells and CSCs, which could trigger apoptotic responses or chemosensitize these cells to other therapies. Furthermore, HDACs affect the activity of non-histone substrates, including HIF-1 and NF-κB, which have been implicated in driving EMT and drug resistance.

### Molecular signaling pathways

#### Hedgehog (Hh) signaling pathway

Previous studies have suggested that the Hh pathway is essential for the maintenance of CSCs in various human cancer types including pancreatic cancer (48), gastric cancer (49) and colorectal cancer (50). It is also responsible for treatment resistance of cancer cells. Thus, inhibitors such as cyclopamine and GDC-0449 (51), that obstruct the Hh signaling pathway, may cause depletion of CSCs, overcome MDR, and enhance the curative effect. Tang *et al* (48) observed that epigallocatechin-3-gallate (EGCG) inhibited the components of the sonic hedgehog (SHh) pathway (SMO, Ptch, Gli1 and Gli2) and Gli transcriptional activity, and the combination of quercetin with EGCG had synergistic inhibitory effects on the self-renewal capacity of CSCs through attenuation of TCF/LEF and Gli activities. The authors suggested that therapeutics targeting the SHh pathway might improve the therapeutic outcome of patients with pancreatic cancer by targeting CSCs.

#### Notch signaling pathway

The Notch signaling pathway plays crucial roles in embryonic development and the proliferation, differentiation and apoptosis of stem cells. Specifically, Notch functions as an oncogenic protein in most human cancers including cervical, lung, colon, head and neck, prostate and pancreatic cancer, while it acts as a tumor suppressor in skin cancer, hepatocellular carcinoma and small cell lung cancer (52).

Fan *et al* (53) used G-secretase inhibitors (GSIs) to block the Notch pathway in glioblastoma, resulting in reduced neurosphere growth and clonogenicity *in vitro*, reduced expression of putative CSC markers and reduced tumor growth *in vivo*. Thus, they suggested that GSIs which block the Notch pathway might be useful chemotherapeutic reagents to target CSCs. MRK-03 has a similar effect on breast cancer stem cells (54).

#### Wnt and other signaling pathways

The Wnt signaling pathway is another developmental pathway involved in multiple biological processes including embryogenesis, development, cell proliferation, survival and differentiation. However, oncogenic mutations or deactivation of the tumor suppressor may result in the dysregulation of the Wnt/β-catenin pathway in CSCs, which induces neoplastic proliferation. Small molecule inhibitors include existing drugs such as nonsteroidal anti-inflammatory drugs (NSAIDs) or natural compounds, and molecular-targeted agents such as the cAMP response-element binding protein (CBP)/β-catenin antagonist ICG-001. These inhibitors interfere with the Wnt pathway and harvest a therapeutic effect. Takahashi-Yanaga *et al* (55) demonstrated that CBP/β-catenin antagonist ICG-001 was able to target and eliminate drug-resistant leukemic stem cells *in vivo* and *in vitro*.

#### Other mechanisms

Other mechanisms are also involved in the MDR of CSCs; for example, microRNAs, which are single-stranded 19–25 nucleotide short RNAs, play a significant role in the regulation of MDR (56). CSCs differentiation-inducing agents also improve the efficacy of treatment in combination with chemotherapy (57).

## 4. Conclusion

Due to poor chemotherapy efficacy in most cancer types, particularly in the case of metastases, we should change our view of the development of drug resistance in cancer and try to develop chemotherapy based on the CSC model. However, there also are certain unresolved issues concerning therapy aimed directly at CSCs. For example, ABC transporters and ALDHs are markers of normal stem cells, and the inhibition of these molecules in CSCs may result in them occurring in normal stem cells, consequently leading to severe side effects. In addition, it is still unclear which ALDH isozymes contribute to the ALDH activity and whether this is the same among all tissue-specific stem cells. It is even claimed that before CSCs can be clearly identified, therapeutic approaches designated to target them may actually cause more harm than good in glioblastoma multiforme patients (58). All these issues remain to be solved in the future.
